# Ligand-driven conformational changes of MurD visualized by paramagnetic NMR

**DOI:** 10.1038/srep16685

**Published:** 2015-11-19

**Authors:** Tomohide Saio, Kenji Ogura, Hiroyuki Kumeta, Yoshihiro Kobashigawa, Kazumi Shimizu, Masashi Yokochi, Kota Kodama, Hiroto Yamaguchi, Hideki Tsujishita, Fuyuhiko Inagaki

**Affiliations:** 1 Department of Structural Biology, Faculty of Advanced Life Science, Hokkaido University, Sapporo 001-0021, Japan; 2 Department of Chemistry, Faculty of Science, Hokkaido University, Sapporo 060-0810, Japan; 3 PRESTO, JST, Tokyo, 135-0063, Japan; 4 Center for Research and Education on Drug Discovery, Faculty of Pharmaceutical Sciences, Hokkaido University, Sapporo 060-0812, Japan; 5 Shionogi Pharmaceutical Research Center, Shionogi & Co., Ltd, Toyonaka 561-0825, Japan; 6 Shionogi Pharmaceutical Research Center, Shionogi & Co., Ltd, Osaka 541-0045, Japan

## Abstract

Proteins, especially multi-domain proteins, often undergo drastic conformational changes upon binding to ligands or by post-translational modifications, which is a key step to regulate their function. However, the detailed mechanisms of such dynamic regulation of the functional processes are poorly understood because of the lack of an efficient tool. We here demonstrate detailed characterization of conformational changes of MurD, a 47 kDa protein enzyme consisting of three domains, by the use of solution NMR equipped with paramagnetic lanthanide probe. Quantitative analysis of pseudocontact shifts has identified a novel conformational state of MurD, named semi-closed conformation, which is found to be the key to understand how MurD regulates the binding of the ligands. The modulation of the affinity coupled with conformational changes accentuates the importance of conformational state to be evaluated in drug design.

Multi-domain proteins undergo drastic conformational changes that are indispensable for their functional process. For example, ligand binding^1^,^2^ or phosphorylation^3^,^4^ triggers conformational changes of the protein to modulate the affinity to another ligand or substrate, which thus regulates catalytic cycles or signal transduction processes. However detailed mechanisms involving conformational changes are poorly understood. Proteins undergoing dynamic conformational changes are difficult to be crystalized, so that some of the important conformations may remain “invisible” in X-ray crystallography^5^. Solution nuclear magnetic resonance (NMR) is one of the best tools to characterize dynamic systems in atomic resolution. The two major limitations in the conventional protein NMR, size limit and the shortage of structural information, are being overcome by the use of isotope labeling and paramagnetic probes^6^.

Here, we report ligand-driven dynamic conformational changes of 47 kDa multi-domain protein MurD as characterized by NMR equipped with paramagnetic lanthanide probe. MurD is one of the ATP-driven Mur ligases that are responsible for peptidoglycan biosynthesis, catalyzing a formation of a peptide bond between the carboxyl group of the UDP-*N*-acetylmuramoyl-l-alanine (UMA) and the amino group of the d-glutamic acid^7^. The crystal structures of MurD^8^,^9^,^10^,^11^ indicate that one of the three domains of MurD, domain 3, undergoes drastic conformational change from open to closed state, which controls the process of the reaction. However the detailed mechanism of its regulation still needs to be elucidated. More specifically, how the substrates (ATP, UMA, and d-Glu) are selected to bind in an appropriate order, and how the binding of the ligands triggers the open-close transition of domain 3 are totally unknown.

To investigate the dynamic structural changes of domain 3 coupled with the enzymatic process, we have exploited paramagnetic lanthanide probe method^6^, where paramagnetic lanthanide ion is fixed in a protein frame by the use of a lanthanide-binding tag^12^,^13^,^14^,^15^ and then long-range structural information is extracted from paramagnetic effects such as pseudocontact shift (PCS). PCS is a chemical shift change induced by paramagnetism of a lanthanide ion and provides long-range (~40 Å) distance and angular information on the observed nuclei. In contrast to the conventional approaches utilizing short-range (~5 Å) distance information derived from nuclear Overhauser effect (NOE), the paramagnetic lanthanide probe provides long-range quantitative information and thus is useful for the rapid structural determination or assessment of protein-protein and protein-ligand complexes^16^,^17^,^18^,^19^,^20^,^21^. The paramagnetic data have identified a novel semi-closed form of MurD as an intermediate in the reaction that has been “invisible” in the reported crystallographic analyses. Combined with NMR titration and isothermal titration calorimetry, the PCS data show that the ligand binding triggers conformational change of MurD that modulates the affinity to the subsequent ligand to regulate the order of the ligand binding.

## Results

### Backbone resonance assignment of MurD

**Figure 1:**
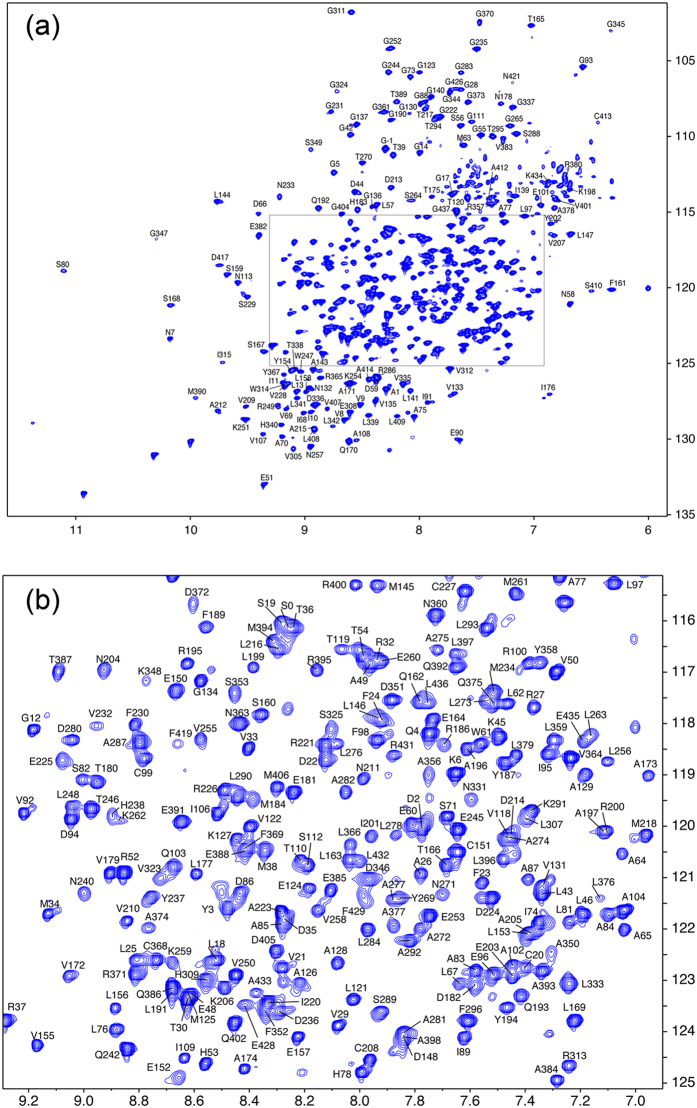
Backbone resonance assignment of ^2^H^13^C^15^N-labeled full-length MurD. (**a**) ^1^H^15^N-HSQC with assignments. (**b**) Expanded view of the region indicated by a box in (**a**).

The NMR resonances of apo *E. coli* MurD were assigned utilizing domain-parsing strategy^3^,^22^,^23^, deuterium labeling, and transverse relaxation-optimized spectroscopy (TROSY)-based NMR experiments^24^. We initially performed backbone signal assignment for domain 1–2 (1–302) and domain 3 (301–437) separately to obtain nearly complete assignment for both domains (*see Methods*). With reference to the resonance assignments for domain 1–2 and domain 3, the backbone resonances of full-length MurD (1–437) were assigned based on the TROSY-based triple-resonance experiments performed on ^2^H/^13^C/^15^N-labeled MurD and almost all of the observed resonances on ^1^H–^15^N HSQC were assigned (Fig. 1). However, a number of resonances from the interface between domain 2 and domain 3 were missing, presumably due to exchange broadening caused by open-close dynamics of domain 3. Note that the isolated domain 1–2 and domain 3 provided uniform resonances for almost all of the backbone amide groups. Addition of the known MurD inhibitors, *N*-({3-[({4-[(*Z*)-(2,4-dioxo-1,3-thiazolidin-5-ylidene)methyl]phenyl}amino)methyl]phenyl}carbonyl)-d-glutamic acid^25^ (compound **1**) and *N*-(6-butoxy-naphthalene-2-sulfonyl)-d-glutamic acid^11^ (compound **2**) (Figure S1) quenched the exchange broadening, resulting in more uniform and intense resonances on the spectrum (Supporting information, Figure S2). This implies that the binding of the inhibitor stabilizes MurD in one conformation. Especially in the presence of the compound **1**, which has higher affinity to MurD than compound **2**, the resonances on the interface became much sharper and even new resonances appeared. We ran a set of TROSY-based triple resonance experiments for MurD in complex with compound **1** and have assigned almost all of the backbone amide resonances (Figures S2 and S3).

### Attachment of a paramagnetic lanthanide ion on MurD

**Figure 2:**
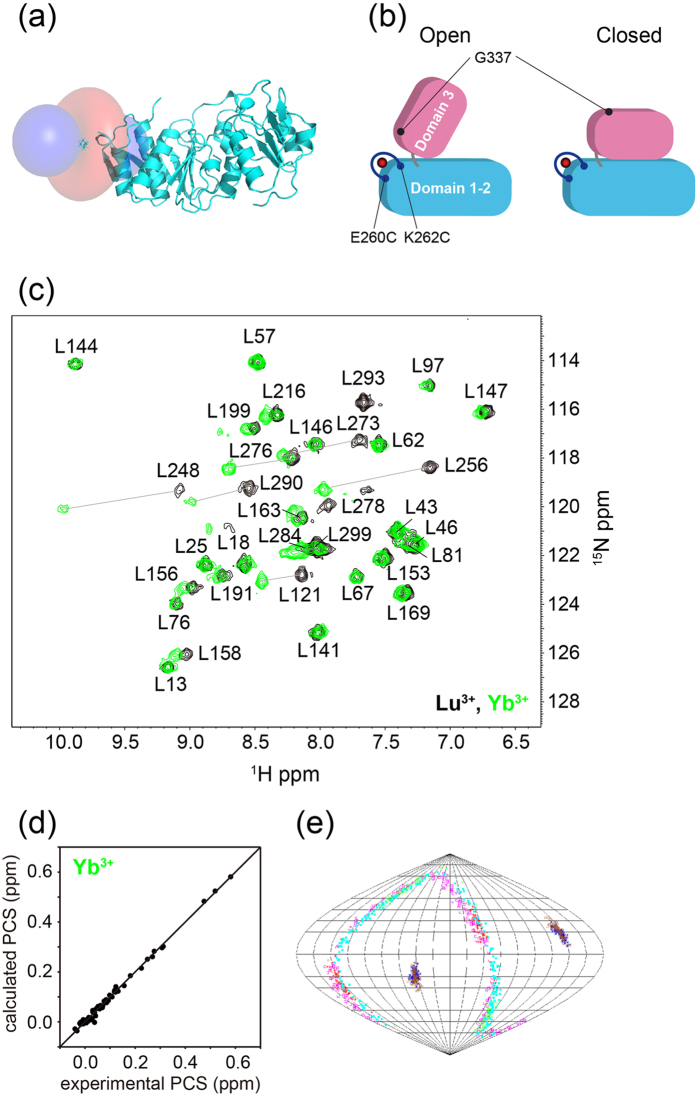
Application of paramagnetic lanthanide probe to MurD for characterization of conformational changes. (**a**) View of experimentally determined PCS isosurfaces depicting the surface corresponding to a PCS of +/− 0.5 ppm induced by Yb^3+^. Positive and negative PCS values are indicated by blue and red, respectively. The structure of domain 1–2 (3uag.pdb)^10^ is shown in cyan. (**b**) Schematic representation of the design of the double Cys-mutation to detect the conformational changes of domain 3 by PCS. Amino acid residues in domain 3 such as G377 are away from the lanthanide ion in the closed conformation but become closer to the ion in the open conformation. (**c**) ^1^H^15^N-HSQC spectra of [^15^N-Leu]-labeled MurD domain 1–2 E260C/K262C attached with CLaNP-5. The spectra in the presence of diamagnetic lanthanide ion Lu^3+^ and paramagnetic lanthanide ion Yb^3+^ are colored in black and green, respectively. (**d**) Comparison of experimental and back-calculated PCSs of backbone amide protons observed for [^15^N-Leu]-, [^15^N-Val]-, [^15^N-Ile]-, or [^15^N-Met]-labeled domain 1–2 containing CLaNP-5 (Yb^3+^). (**e**) Orientation of the principal axes of the Δ*χ*-tensors of Yb^3+^ and Tm^3+^, in complex with CLaNP-5 fixed on MurD domain 1–2, visualized in Sanson-Flamsteed projection. The plots show the points where the principal axes of the Δ*χ*-tensor penetrate the sphere. One hundred sets of plots represent the results of the Monte-Carlo analysis using the 100 partial PCS data sets in which 30% of the input data were randomly deleted.

The NMR spectra of apo MurD implied that domain 3 undergoes dynamic conformational changes. Previous studies have also suggested drastic conformational changes of domain 3 during the catalytic process^7^,^8^,^10^,^26^. In order to investigate the conformational changes of MurD in solution, we exploited the paramagnetic lanthanide probe method that provides long-range (~40 Å) quantitative distance and angular information. Paramagnetic lanthanide ion, Yb^3+^ or Tm^3+^, or diamagnetic lanthanide ion Lu^3+^ was fixed on domain 2 by a lanthanide-binding tag: Caged Lanthanide NMR Probe 5 (CLaNP-5)^14^,^15^. Based on the crystal structures of MurD (1e0d.pdb and 3uag.pdb)^8^,^10^, two exposed residues, E260 and K262, were selected for the anchoring points for CLaNP-5 so that the largest changes of the paramagnetic effects can be detected upon the conformational changes of domain 3 (Fig. 2b). Two constructs were designed: full-length MurD containing E260C/K262C mutations and MurD domain 1–2 containing E260C/K262C mutations. Although MurD has 7 cysteines, all of them are buried: CLaNP-5 didn’t react with wild type MurD as confirmed by NMR (data not shown). For the determination of anisotropic magnetic susceptibility tensor, Δ*χ*-tensor, we prepared the [^15^N-Leu]-, [^15^N-Val]-, [^15^N-Ile]-, or [^15^N-Met]-labeled domain 1–2 containing CLaNP-5 (Lu^3+^), CLaNP-5 (Yb^3+^), or CLaNP-5 (Tm^3+^) and measured ^1^H^15^N-HSQC spectra (Fig. 2c). 32 and 31 PCSs greater than 0.05 ppm induced by Yb^3+^ and Tm^3+^, respectively, were observed from amide protons (Table S1). Δ*χ*-tensor was calculated by Numbat program^27^, based on the PCSs observed for domain 1–2 with CLaNP-5 and crystal structure of MurD (3uag.pdb)^10^ (Table 1, Fig. 2a,d,e). The tensor values are close to those determined in the previous works^14^,^15^ and the correlation between calculated and observed PCS values is very good, indicating that the tag was successfully introduced on MurD. It should be noted that the orientations of x- and y-axis were not well defined (Fig. 2e), because the tensor has axial symmetry due to the symmetric structure of the CLaNP-5^14^,^15^.

### PCS-based characterization of ligand-driven conformational changes of MurD

**Figure 3:**
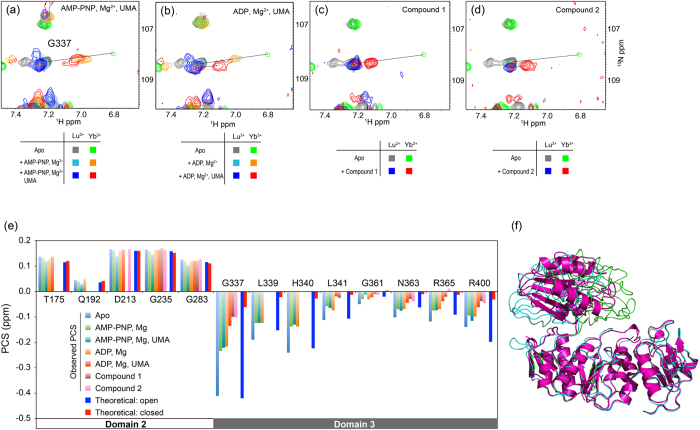
PCS analysis for full-length MurD. (**a–d**) Overlay of ^1^H^15^N-HSQC spectra of ^15^N MurD E260C/K262C attached with CLaNP-5 containing Lu^3+^ (diamagnetic) or Yb^3+^ (paramagnetic), titrated with a series of the ligands. Chemical shift difference between paramagnetic (Yb^3+^) and diamagnetic (Lu^3+^) state is PCS that is indicated by a black line. (**e**) PCSs observed for the backbone amide proton resonances from domain 2 and 3 in the presence or absence of the ligands. Theoretical values for open and closed state are also displayed. While the PCSs observed from domain 2 don’t change in all of the conditions, the PCSs from domain 3 drastically change depending on the combination of the ligand. (**f**) Model of semi-closed model (purple) calculated based on PCSs. The model is superimposed with crystal structures of MurD in open (1e0d.pdb, cyan)^8^ and closed (3uag.pdb, green)^10^ conformations.

Yb^3+^-induced PCSs were observed for ^15^N-labeled full-length MurD in various conditions. The largest PCS change upon the ligand binding was observed for G337 (Figs 2b and 3a–d). Other resonances from domain 3 also indicated large PCS changes, while the PCSs from domain 2 didn’t change in all of the conditions, proving that the structure of domain 2 and paramagnetic effects of the lanthanide ion remain identical throughout the experiments (Fig. 3e). In comparison with the theoretical values, PCSs observed for apo MurD well matched to the theoretical values for open state, suggesting that apo MurD in solution has on average open state as observed in the crystal structure (1e0d.pdb)^8^. The theoretical PCS values for full-length MurD were calculated based on the crystal structure of open state (1e0d.pdb)^8^ or closed state (3uag.pdb)^10^ of MurD and the tensor parameters fitted based on the PCSs observed for isolated domain1–2. Perfect matching of the observed PCSs for the residues in domain 2 in full-length MurD to the theoretical values proves that the tensor determined for isolated domain 1–2 is preserved in full-length MurD. Since the tensor is determined by local environment such as coordination and mobility of the tag against the protein, it is quite reasonable to have the same tensor in both of isolated domain 1–2 and full-length MurD. Binding of ATP analogue adenylylimidodiphosphate (AMP-PNP) and Mg^2+^ resulted in drastic decrease of the magnitude of PCSs from domain 3 (Fig. 3a,e), but the PCSs were not small enough compared to the theoretical values for the closed state, rather they were in between open and closed state. Further addition of the ligands didn’t change the PCSs, proving that the binding was saturated (data not shown). These observations implied the existence of another conformational state of MurD that has been veiled in the previous crystallographic studies. Here we name it “semi-closed” state and calculated a model based on PCSs observed for MurD in complex with AMP-PNP and Mg^2+^ (Fig. 3f). As expected from the PCS values, domain 3 is located at the midpoint between open and closed state.

Addition of ADP and Mg^2+^ to MurD exhibited PCSs similar to those induced by AMP-PNP and Mg^2+^. However, different PCS profiles were observed in the presence of UMA: Addition of UMA didn’t cause further PCS change in the presence of AMP-PNP and Mg^2+^, while addition of UMA in the presence of ADP and Mg^2+^ induced further decrease in the magnitude of PCSs, resulting in the PCSs very close to the theoretical values for closed state (Fig. 3a,b,e). This observation implied that the hydrolysis of ATP triggers further conformational change from semi-closed to fully closed state. The PCSs observed in the presence of compound **1** or **2** were close to those observed in the presence of ADP, Mg^2+^, and UMA, as well as theoretical values for closed form of MurD (Fig. 3c–e), suggesting that binding of these ligands triggers MurD in closed conformation as observed in the crystal structure (3uag.pdb)^10^.

### Interaction between MurD and its ligands monitored by NMR and ITC

**Figure 4:**
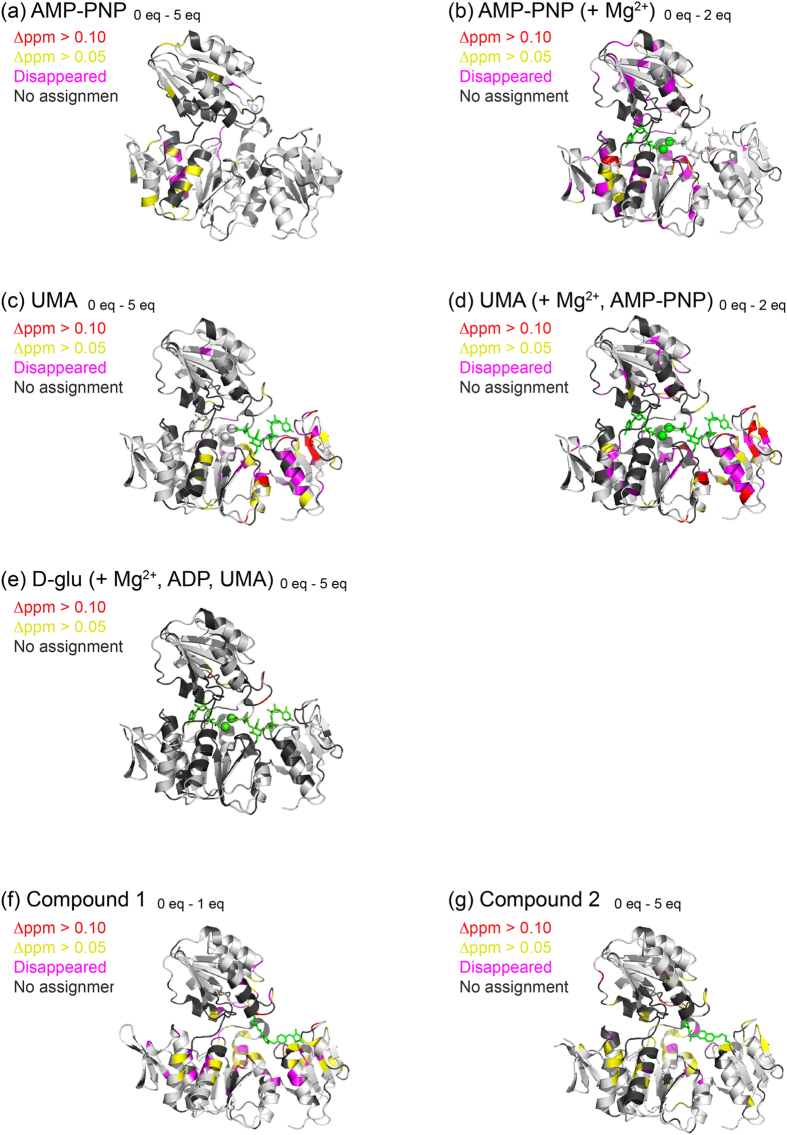
Mapping of the chemical shift perturbations upon ligand binding. The chemical shift perturbation observed in the backbone amide groups of MurD by the addition of its ligands. (**a**) Perturbation by the addition of 5 equivalents of AMP-PNP. (**b**) Perturbation by the addition of 2 equivalents of AMP-PNP in the presence of 20 equivalents of Mg^2+^. (**c**) Perturbation by the addition of 5 equivalents of UMA. (**d**) Perturbation by the addition of 2 equivalents of UMA in the presence of 20 equivalents of Mg^2+^ and 3 equivalents of AMP-PNP. (**e**) Perturbation by the addition of 5 equivalents of d-Glu in the presence of 20 equivalents of Mg^2+^, 5 equivalents of AMP-PNP, and 3 equivalents of UMA. (f) Perturbation by the addition of 1 equivalent of compound **1**. (**g**) Perturbation by the addition of 5 equivalent of compound **2**.

In order to investigate the ligand bindings, several sets of HSQC-based titration experiments were conducted for MurD and its ligands: Mg^2+^, AMP-PNP, UMA, d-Glu, compound **1**, and compound **2** (Figs 4 and S2). The addition of AMP-PNP, UMA, compound **1**, and compound **2** resulted in the significant perturbations at large region in MurD, suggesting conformational changes upon the ligand binding (Fig. 4). The binding of AMP-PNP was fast in NMR time scale, but became slow in the presence of Mg^2+^, indicating that the binding affinity of ATP became stronger by the presence of Mg^2+^. Likewise, the binding of UMA became stronger in the presence of AMP-PNP and Mg^2+^. The final ligand d-Glu interacted with neither apo MurD (data not shown) nor MurD in complex with AMP-PNP, Mg^2+^, and UMA (Figure S2f). d-Glu bound to MurD only in the presence of ADP, Mg^2+^, and UMA, suggesting that the ATP hydrolysis allows d-Glu to bind to MurD. This observation was further corroborated by ITC experiments where the binding between d-Glu and MurD was seen in the presence of ADP, Mg^2+^, and UMA, but not in the presence of AMP-PNP, Mg^2+^, and UMA (data not shown). Dissociation constants were determined based on the chemical shift changes or peak volume changes upon the addition of the ligands at several conditions (Table 2). The dissociation constants also indicate affinity enhancement caused by the binding of the other ligands. HSQC-based titrations were further corroborated by isothermal titration calorimetry (ITC). As described in Table 2 and Figure S5, dissociation constants determined by ITC have good agreement with those determined by NMR.

## Discussion

### Apo MurD exhibits dynamic open-close exchange that is stabilized by inhibitors

During the assignment of full-length MurD, we found that a number of resonances from the interface between domain 2 and 3 were missing due to exchange broadening, which was suppressed by the addition of compound **1** or **2** (Figures S2h,i, and S4). Given the fact that compound **1** and **2** induce the closed conformation (Fig. 3), this observation implies that apo MurD has open-close exchange in μs-ms timescale, but the binding of the ligand stabilizes MurD in the closed conformation as observed in the crystal structures (2 × 5o.pdb and 2jff.pdb)^11^,^25^. We have assigned almost all of the backbone amide resonances of MurD in the presence of compound **1** (Figure S3).

### Substrate binding enhances the affinity for the subsequent substrate

We investigated the affinities between MurD and its ligands by NMR and ITC. The data from NMR and ITC are compatible with each other, both supporting the idea that the affinity for one ligand is enhanced by the preceding ligands. As shown in Table 2 and Figure S2, UMA has stronger affinity to MurD in the presence of AMP-PNP and Mg^2+^. Affinity for d-Glu is also increased by the presence of UMA, ADP, and Mg^2+^, but, interestingly, not by UMA, AMP-PNP, and Mg^2+^ (Figure S2f,g). This suggests that hydrolysis of ATP cause further structural change that allows d-Glu to bind to MurD. Our experiments clearly demonstrate the order of the ligand binding to MurD: ATP binds first in the presence of Mg^2+^, followed by UMA, ATP hydrolysis, and finally d-Glu, which is consistent with the previous studies on MurC and MurF^28^,^29^.

### Dynamic conformational changes of MurD modulate the affinity, thus determine the order of the substrate binding

However, how does MurD control the affinity to the ligands? We address this question by paramagnetic lanthanide probe. As shown in Fig. 4, addition of AMP-PNP or UMA affected to the large area of MurD, implying the significant conformational changes triggered by ligand binding. Since MurD is thought to undergo drastic conformational change of domain 3, from open to closed conformation during its catalytic process, the orientation of domain 3 was investigated based on PCS. The lanthanide-binding tag, CLaNP-5^14^,^15^, was attached *via* two cysteine mutations at E260 and K262 located on the edge of domain 2 (Fig. 2a and b). PCS depends on the distance and angle of the observed nuclei against paramagnetic ion, and can be detected up to 40 Å. Thus, the orientation of domain 3 can be visualized by PCSs observed for domain 3. As shown in Fig. 3, the resonances from domain 3 in apo MurD indicated large PCSs that match to the theoretical values calculated based on the crystal structure of the open form of MurD (1e0d.pdb)^8^, suggesting that apo MurD has domain 3 in the same orientation as observed in the crystal structure.

The resonances from domain 3 exhibited drastic PCS changes upon the addition of AMP-PNP and Mg^2+^, or ADP and Mg^2+^ (Fig. 3). PCSs observed under these conditions correspond to neither the theoretical value of the open form nor closed form (Fig. 3e), indicating that the MurD is in “semi-closed” conformation where domain 3 is located in between open and closed conformation (Fig. 3f). In the crystal structure of fully closed MurD in complex with UMA, ADP, and Mg^2+^ (3uag.pdb)^10^, there is not enough space for γ-phosphate, implying steric clash between γ-phosphate group of ATP and ε-amino group of Lys319 (Figure S6). This possible steric clash may hold MurD in semi-closed conformation where Lys319 is located away to make a space for the γ-phosphate group. Hydrolysis of ATP into ADP allows Lys319 to come closer to UMA and ADP, forming hydrogen bonds (Figure S6). Despite a number of crystal structures reported in the absence or presence of various ligands, they have either open or closed conformation and none of them satisfies PCSs observed for MurD in complex with AMP-PNP and Mg^2+^, with AMP-PNP, Mg^2+^, and UMA, or with ADP and Mg^2+^. Interestingly, no crystal structure has been reported for MurD in complex with ATP or its analogue. Bertrand *et al.* reported that AMP-PNP was hydrolyzed during crystallization^10^, suggesting that the ATP-bound form of MurD, semi-closed form, is unfavorable for crystallization, which has obscured the intermediate state for a long time. Addition of UMA in the presence of ADP and Mg^2+^ causes further changes of PCSs, while PCSs don’t change by the addition of UMA in the presence of AMP-PNP and Mg^2+^ (Fig. 3). The PCS values of MurD in complex with ADP, Mg^2+^, and UMA are very close to the theoretical values for the closed form (3uag.pdb)^10^. Combined with the fact that d-Glu binds to MurD only in the presence of ADP, Mg^2+^, and UMA (Figure S2f,g), this result suggests that hydrolysis of ATP into ADP triggers conformational change from semi-closed to fully closed form, which allows MurD to bind to the final substrate d-Glu. This idea is consistent with the fact that the residues from both of domain 2 (D182) and domain 3 (T321, K348, S415, and F422) are involved in the interaction with d-Glu^10^. D182 is separated away from the other residues in semi-closed conformation, which presumably reduces the affinity to d-Glu. Only in the fully closed conformation, d-Glu is properly fixed on MurD, which allows final nucleophilic attack of the nitrogen in d-Glu to carbonyl in UMA followed by SN2 displacement of the phosphate as proposed by Bertrand *et al.*^10^.

**Figure 5:**
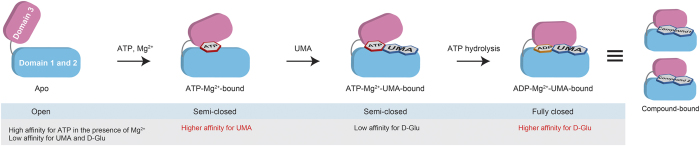
Summary of conformational changes of MurD that modulate the affinities for the ligands. Apo MurD, with domain 3 existing in open state, has high affinity for ATP-Mg^2+^ but has low affinity for UMA or d-Glu. Binding of ATP-Mg^2+^ triggers conformational change of MurD to semi-closed conformation where the domain 3 is located in between open and closed state. Semi-closed state has higher affinity for UMA, but still has low affinity for d-Glu. Binding of UMA doesn’t change the conformation, but hydrolysis of ATP into ADP allows MurD to fully closed conformation that has higher affinity for d-Glu.

Figure 5 summarizes the conformational changes of MurD coupled with its enzymatic reaction. Although apo MurD exists in open-close equilibrium as indicated by exchange broadening, the open form is the major component as indicated by PCS analysis. Apo MurD in open conformation has lower affinity for UMA or d-Glu, but has higher affinity for ATP in the presence of Mg^2+^ (Table 2). Upon the binding to ATP-Mg^2+^, MurD forms semi-closed conformation to acquire stronger affinity to UMA but still not to d-Glu. Thus MurD selectively binds to the second substrate UMA. After the binding to UMA, MurD still has semi-closed conformation and has lower affinity for d-Glu, but hydrolysis of ATP triggers conformational change of domain 3 to fully closed form where the third substrate d-Glu binds. Previously reported drugs, compound **1** and **2**, also fix the conformation in the fully closed form, thus block any substrate binding to MurD.

Given the important role of MurD in peptidoglycan synthesis in bacteria, MurD is one of the best possible targets for antibiotic drugs, and in fact several compounds targeting to MurD have been reported to date^11^,^25^,^30^,^31^. One of the strongest strategies for drug design is fragment based drug design (FBDD) where small simple compounds (fragments) are screened for binding to a target protein, and the hit compounds are then combined and modified to increase the affinity. In FBDD, it is inevitable to evaluate the binding property for each of the hits as well as to obtain structural information. As shown above, however, MurD has different affinities even to the same ligand, modulated by other ligand coupled with dynamic conformational changes of domain 3. Here our study highlights the importance of conformational states to be evaluated along with ligand binding, in drug discovery for multi-domain proteins.

### Online methods

#### Preparation of CLaNP-5

CLaNP-5 was synthesized, purified, and chelated to Lu^3+^, Yb^3+^, and Tm^3+^, according to the procedure reported by Keizers *et al.*^14^,^15^. Each CLaNP-5 complex was purified using a preparative C-18 reversed phase column (Inertsil ODS-3 20 mm × 250 mm) on a SHIMADZU liquid chromatography system (HPLC) with an LC-6AD pump, at a flow rate of 7 mL/min. The column temperature was 25 °C, and UV monitoring was carried out at 254 nm. Solvent A was distilled water containing 0.1% TFA, and solvent B was acetonitrile containing 0.1% TFA. A linear gradient of 5–35% of B over 45 min was used.

#### Preparation of the protein samples for NMR resonance assignment and HSQC based titration experiments

*E. coli* MurD full-length (1–437), domain 1–2 (1–302), and domain 3 (301–437) were cloned into pGBHPS^32^ and expressed in *E. coli* strain BL21 (DE3). For the preparation of ^15^N- or ^13^C^15^N-labeled protein, cells were grown in M9 minimal media containing ^15^NH_4_Cl (1 g/L), Celtone-N or Celtone-CN powder (0.2 g/L) (Cambridge Isotope Laboratories, USA) and ^12^C- or ^13^C-glucose (2 g/L). Cells were grown at 37 °C to A_600_ of 0.8, and protein expression was induced by the addition of isopropyl β-d-1-thiogalactopyranoside (IPTG) to a final concentration of 0.5 mM for 16 h at 25 ^O^C. All of the protein samples were purified using Ni-NTA resin (QUIAGEN, UK), followed by the tag removal with HRV3C protease. The isolated protein was further purified by gel filtration chromatography on a Superdex 75 column (GE Healthcare) equilibrated with 50 mM Tris-HCl (pH 8.0), 500 mM NaCl. For the preparation of ^2^H^13^C^15^N-labeled protein, cells were grown in 100% ^2^H_2_O M9 minimal media containing ^15^NH_4_Cl (1 g/L), Celtone-DCN powder (0.2 g/L) (Cambridge Isotope Laboratories, USA) and ^2^H^3^C-glucose (2 g/L). In order to exchange ^2^H at the amide group into ^1^H, the protein was unfolded by 6 M guanidine hydrochloride in 50 mM Tris-HCl (pH 8.0), 150 mM NaCl, 1 mM dithiothreitol (DTT), followed by refolding using dialysis against 50 mM Tris-HCl (pH 8.0), 150 mM NaCl, 1 mM DTT at 4 °C for 16 h. The refolded protein was purified by gel filtration chromatography. For the NMR experiments of domain 1–2 and domain 3, the buffer was exchanged into 20 mM Tris-HCl (pH 7.2), 100 mM NaCl, 10 mM DTT. For the NMR experiments of full-length MurD, the buffer was exchanged into 20 mM Tris-HCl (pH 7.2), 200 mM NaCl, 10 mM DTT. Typically 1L of M9 medium yielded 35 mg of MurD.

#### Preparation of the protein samples for PCS observation

For PCS observation, MurD full-length and domain 1–2 both containing E260C and K262C mutations were cloned into pGBHPS^32^ and expressed in *E. coli* BL21 (DE3) cells. For the preparation of uniformly ^15^N-labeled samples, the *E. coli* cells are grown in M9 minimal media as described above. For the preparation of amino acid selectively ^15^N-labeled samples, the cells were grown at 37 °C in 1L of minimal media supplemented with 1 g ^14^NH_4_Cl and 200 mg of 19 unlabeled amino acids. 50 mg of specific ^15^N-labeled amino acid was added to the medium 15 min before the expression induction. Cells were incubated for 3 h at 37 °C after the induction. The protein samples were purified as described above. 10 mM DTT was added to the sample before gel filtration. After gel filtration, the sample was mixed with 1 equivalent of CLaNP-5 containing Lu^3+^, Yb^3+^, or Tm^3+^. The mixture was incubated on ice for 15 minutes, followed by gel filtration. For the NMR experiments of domain 1–2 E260C/K262C containing CLaNP-5, the buffer was exchanged into 20 mM Tris-HCl (pH 7.2), 100 mM NaCl. For the NMR experiments of full-length MurD E260C/K262C, the buffer was exchanged into 20 mM Tris-HCl (pH 7.2), 200 mM NaCl.

#### NMR measurement and resonance assignment

NMR experiments were run on UNITY inova 800 or 600 MHz NMR spectrometers (Agilent, USA). 3D spectra for resonance assignment of full-length MurD were recorded at 35 °C, and all other spectra were recorded at 25 °C. Spectra were processed using the NMRPipe program^33^ and data analysis was performed with the help of the Olivia program developed in our laboratory (Yokochi *et al.* http://fermi.pharm.hokudai.ac.jp/olivia/). Backbone resonance assignments for ^2^H^13^C^15^N-labeled MurD full-length and domain 1–2 were carried out using the following set of spectra; ^1^H–^15^N TROSY-HSQC, TROSY-HN(CO)CA, TROSY-HNCA, TROSY-HNCACB, TROSY-HNCO, TROSY-HN(COCA)CB, and ^15^N-edited NOESY-HSQC. 96% of backbone amide resonances of domain 1–2 were assigned. Backbone resonance assignment for ^13^C^15^N-labeled MurD domain 3 was carried out using the following set of spectra; ^1^H–^15^N HSQC, HN(CO)CA, HNCA, HNCACB, CBCA(CO)NH, HNCO, and ^15^N-edited NOESY-HSQC. 96% of backbone amide resonances were assigned. With the reference of the assignments for isolated domains, the resonances of full-length MurD were assigned to obtain 88% and 97% completeness for apo MurD and MurD in complex with compound 1, respectively.

#### ITC measurement

Isothermal titration calorimetry (ITC) measurements were performed on a MicroCal Auto-iTC200 calorimeter (GE Healthcare). MurD protein samples were extensively dialyzed in phosphate-buffered saline (pH 7.4), and the ligand samples were dissolved in the same dialysis buffer. The following ligand samples were titrated into MurD: AMP-PNP, AMP-PNP in the presence of 5 mM MgCl_2_, UMA, UMA in the presence of 2 mM AMP-PNP and 5 mM MgCl_2_, and compound **1**. For experiments of UMA and compound **1**, DMSO was added to each solution at the final concentration of 5%. All experiments were carried out at 30 °C. The titration of ligand samples (2.0 mM each) into MurD (0.18 mM) were conducted with 20 injections, beginning with a single 0.4 μL injection, and the remaining injections of 2 μL each, with intervals of 150 s. The blank data obtained by titration of the ligands into the buffer were subtracted from the actual titration data after the first titration point was removed. The binding constants were calculated using MicroCal Origin software by nonlinear least squares fitting to one-site binding model.

#### Δχ-tensor calculation

Δ*χ*-tensor and the position of the lanthanide ion were calculated from the PCS values and the crystal structure of MurD (3uag.pdb)^10^, using the Numbat program^27^ with equation Eq. (1),


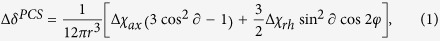


where Δδ^*PCS*^ is the pseudocontact shift, *r*, ∂ and φ are polar coordinates of the nucleus with respect to the principal axes of the magnetic susceptibility tensor, and Δ*χ*_*ax*_ and Δ*χ*_*rh*_ are the axial and rhombic components, respectively, of the anisotropic magnetic susceptibility tensor.

#### Calculation of semi-closed model

A model of semi-closed MurD was calculated by Xplor-NIH^34^,^35^ equipped with PARA restraints for Xplor-NIH^36^, based on Yb^3+^-induced PCSs observed for MurD in complex with AMP-PNP and Mg^2+^. Δ*χ*-tensor was fixed to that determined for domain 1–2. The calculations were started from closed conformation of MurD (3uag.pdb)^10^. In the calculation, domain 1–2 and domain 3 were kept rigid while the linker between domain 2 and domain 3 was set flexible.

## Additional Information

**How to cite this article**: Saio, T. *et al.* Ligand-driven conformational changes of MurD visualized by paramagnetic NMR. *Sci. Rep.* **5**, 16685; doi: 10.1038/srep16685 (2015).

**BMRB accession number**:The resonances assignments have beed deposited to BMRB (code: 26641).

## Supplementary Material

Supplementary Information

## Figures and Tables

**Table 1:** Magnetic susceptibility tensors of lanthanide ions attached to MurD domain 1–2 E260C/K262C.

Lanthanide	Δ*χ*_ax_^a^	Δ*χ*_rh_^a^	α^b^	β^b^	γ^b^
Yb^3+^	8.45 ± 0.46	0.56 ± 0.70	137.0	69.3	22.5
Tm^3+^	47.01 ± 2.30	8.48 ± 5.18	137.0	72.2	59.1

^a^Δ*χ*_ax_ and Δ*χ*_rh_ values are in 10^−32^ [m^3^] and error estimates were obtained by Monte-Carlo protocol using the 100 partial PCS data sets in which 30% of the input data were randomly deleted.

^b^The Euler angles (α, β, γ) are represented in ZYZ convention in degrees.

**Table 2:** Binding affinities for MurD ligands.

Ligand	Condition	Kd [μM]	
		NMR	ITC
Mg^2+^		ND^a^	–
AMP-PNP		630 ±40	300 ± 75
AMP-PNP	Mg^2+^	8.4 ± 1.1	19.8 ±1.7
UMA		930 ± 30	–
UMA	Mg^2+^, AMP-PNP	28.5 ± 5.0	62.9 ± 4.7
d-Glu		ND^a^	–
d-Glu	Mg^2+^, AMP-PNP, UMA	ND^a^	–
d-Glu	Mg^2+^, ADP, UMA	–	–
Compound **1**		8.9 ± 2.4	–
Compound **2**		110 ± 17	116 ± 10

^a^Not detected: Binding was too weak to be detected.
