# Supplementary material for: Ligand-driven conformational changes of MurD visualized by paramagnetic NMR
Source: Sci Rep. 2015 Nov 19;5:16685. doi: 10.1038/srep16685 (PMC4652230; doi:10.1038/srep16685)
Supplement: Supplementary Information [file srep16685-s1.pdf]

Supporting information for

Ligand-driven conformational changes of MurD visualized by paramagnetic NMR

Tomohide Saio, Kenji Ogura, Hiroyuki Kumeta, Yoshihiro Kobashigawa, Kazumi Shimizu, Masashi Yokochi, Kota Kodama, Hiroto Yamaguchi, Hideki Tsujishita and Fuyuhiko Inagaki

Table S1 PCS values observed for MurD domain 1-2 attached with CLaNP-5 containing Yb^{3+} or Tm^{3+}.

	Yb^{3+}		Tm^{3+}	
	HN	N	HN	N
VAL8	0.010	-0.019	0.017	-0.019
VAL9	0.000	-0.007	0.013	0.017
ILE10	0.013	0.035	0.016	-0.052
ILE11	0.015	-0.014	0.002	-0.016
LEU13	-0.003	0.039	-0.028	-0.067
LEU18	-0.013	-0.031		
VAL21	-0.017	-0.033		
LEU25	0.005	-0.006	-0.009	-0.022
VAL29	0.002	0.002	0.025	0.092
VAL33	0.002	-0.016	-0.009	-0.023
MET34	0.006	0.004	0.001	-0.044
MET38			-0.004	-0.024
LEU43	-0.007	-0.012	-0.039	-0.099
LEU46	-0.018	-0.009		
VAL50	-0.021	-0.005	-0.032	-0.058
LEU57	0.005	-0.017	0.023	-0.043
LEU62	0.009	0.014	0.028	0.011
MET63	0.009	0.009	0.067	0.032
LEU67	0.010	0.011	0.050	0.069
ILE68	0.028	0.033	0.088	0.150
VAL69	0.029	-0.002	0.049	0.079
ILE74	0.019	0.012	0.081	0.027
LEU76	0.032	-0.001	0.085	0.068
LEU81	0.021	0.031		
ILE89	0.010	0.012	0.015	0.000
ILE91	0.024	0.030	0.101	0.150
VAL92	0.017	0.050	0.104	0.112
ILE95	0.042	0.073	0.144	0.125
LEU97	0.015	-0.037	0.165	0.056
ILE106	0.058	0.113	0.329	0.383
VAL107	0.086	0.018		
ILE109	0.108	0.110	0.600	0.544
VAL118	0.249	0.277		
LEU121	0.312	0.313		
VAL122	0.276	0.279		
VAL131	-0.032	-0.075		
VAL133	-0.040	-0.033		
VAL135	-0.005	-0.001		
ILE139	0.042	0.040	0.212	0.158
LEU141	-0.010	-0.033		
LEU144	-0.007	0.003		
MET145	-0.003	0.002		
LEU146	-0.003	0.011		
LEU147	0.038	0.033		
LEU153	0.017	0.032		
VAL155	0.045	0.047		
LEU156	0.085	0.097	0.492	0.406
LEU158	0.093	0.025	0.487	0.323
LEU163	0.031	0.016	0.259	0.272
LEU169	0.038	0.016	0.233	0.259
VAL172	0.062	0.093	0.412	0.429
ILE176	0.132	0.162	0.699	0.664
LEU177	0.123	0.117	0.737	0.633
VAL179	0.098	0.057	0.563	0.527
MET184			0.165	0.212
LEU191	0.031	0.030	0.222	0.213
LEU199	0.052	0.030	0.319	0.281
ILE201	0.071	0.035	0.351	0.255
VAL207	0.076	0.024		
VAL209	0.118	0.094		
VAL210	0.156	0.217	0.855	0.799
LEU216	0.088	0.066	0.489	0.447
MET218	0.084	0.088	0.484	0.423
ILE220	0.069	0.099	0.312	0.287
VAL228	0.121	0.105	0.677	0.599
VAL232	0.225	0.255	1.181	1.399
MET234	0.181	0.191	1.022	0.949
LEU248	0.886	0.826	1.978	1.237
VAL250	0.307	0.429		
VAL255	0.519	0.519		
LEU256	0.827	0.919		
LEU273	0.581	0.668		
LEU276	0.475	0.439		
LEU278	0.269	0.281		
LEU284	0.117	0.074		
LEU290	0.440	0.526		

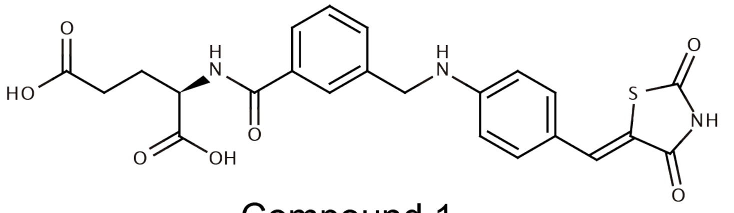

Compound 1

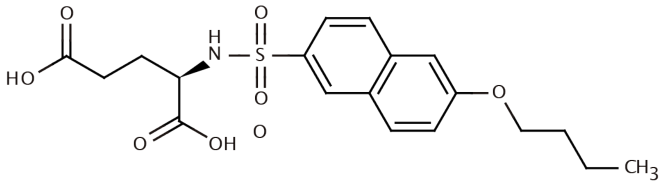

Compound 2

Figure S1 MurD inhibitors used in this study. Compound **1**: *N*-({3-[({4-[(*Z*)-(2,4-Dioxo-1,3-thiazolidin-5-ylidene)methyl]phenyl}amino)methyl]phenyl}carbonyl)-D-glutamic acid. Compound **2**: *N*-(6-Butoxy-naphthalene-2-sulfonyl)-D-glutamic acid.

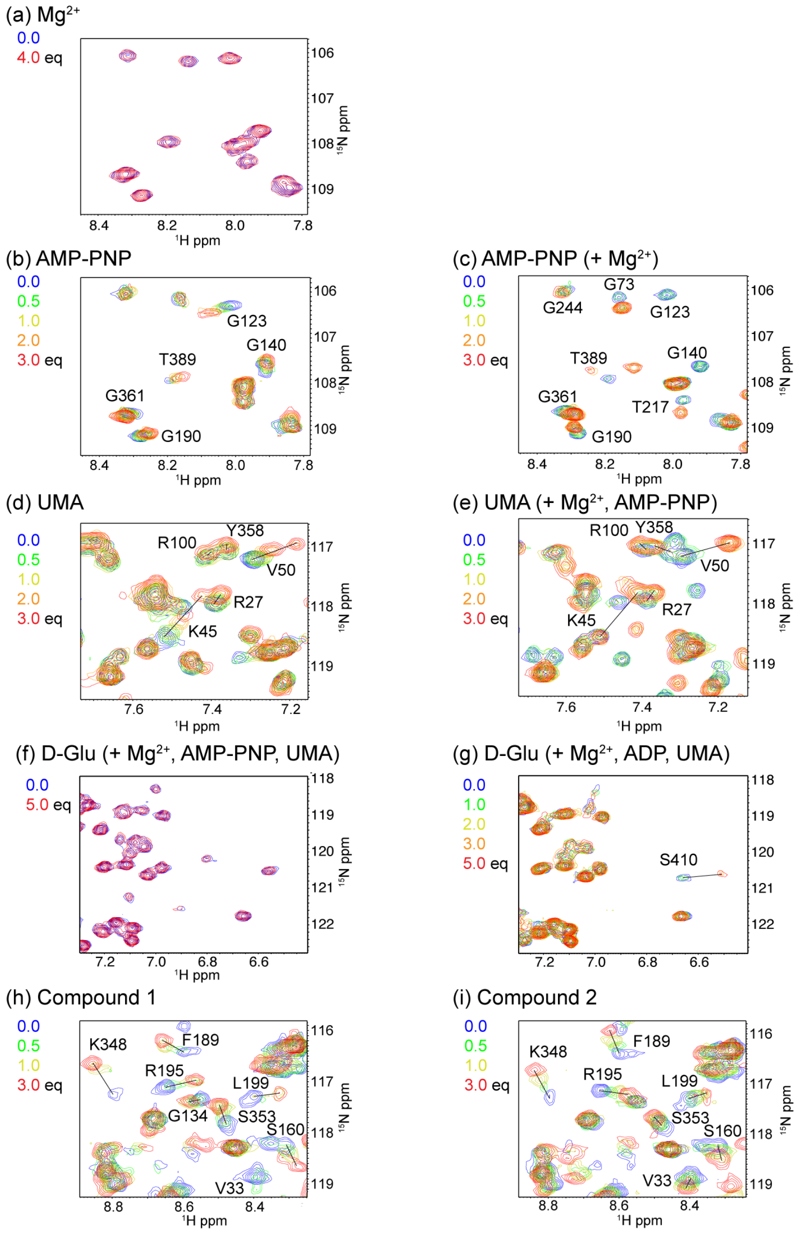

Figure S2 NMR titration experiments for full length MurD with its various ligands. Selected regions of $^{1}H^{15}N$-HSQC spectra of ^{15}N MurD acquired in the presence of increasing amount of the ligands. (a) Titration with Mg^{2+}. (b) Titration with AMP-PNP. (c) Titration with AMP-PNP in the presence of 20 equivalents of Mg^{2+}. (d) Titration with UMA. (e) Titration with UMA in the presence of 20 equivalents of Mg^{2+} and 3 equivalents of AMP-PNP. (f) Titration with D-Glu in the presence of 20 equivalents of Mg^{2+}, 5 equivalents of AMP-PNP, and 3 equivalents of UMA. (g) Titration with D-Glu in the presence of 20 equivalents of Mg^{2+}, 5 equivalents of ADP, and 3 equivalents of UMA. (h) Titration with compound **1**. (i) Titration with compound **2**.

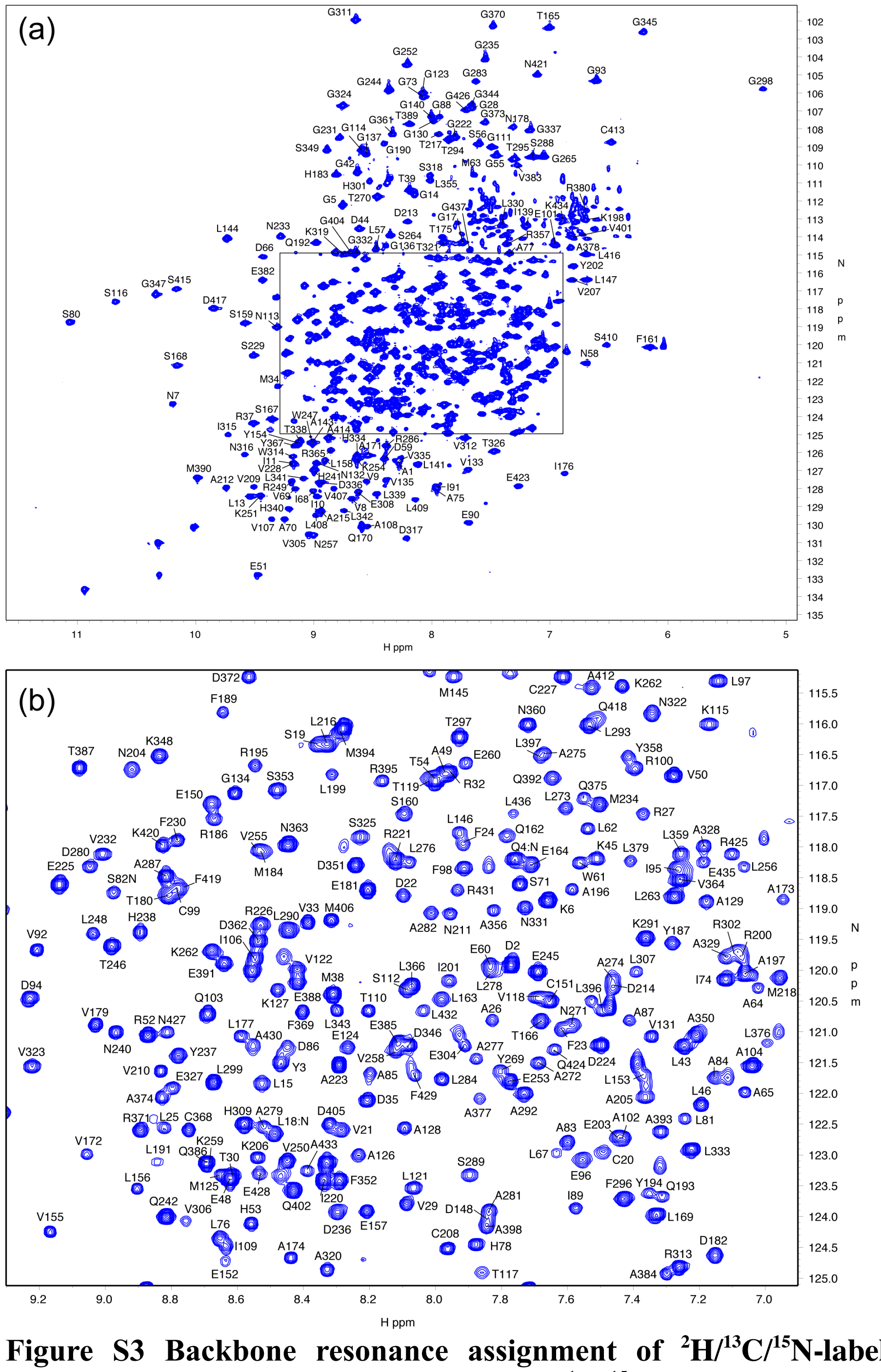

Figure S3 Backbone resonance assignment of $^{2}H/^{13}C/^{15}N$-labeled full length MurD in complex with compound 1. (a) ^{1}H-^{15}N HSQC spectrum of MurD in the presence of 5 equivalents of compound 1 with assignments. (b) Expanded view of the region indicated by a box in (a).

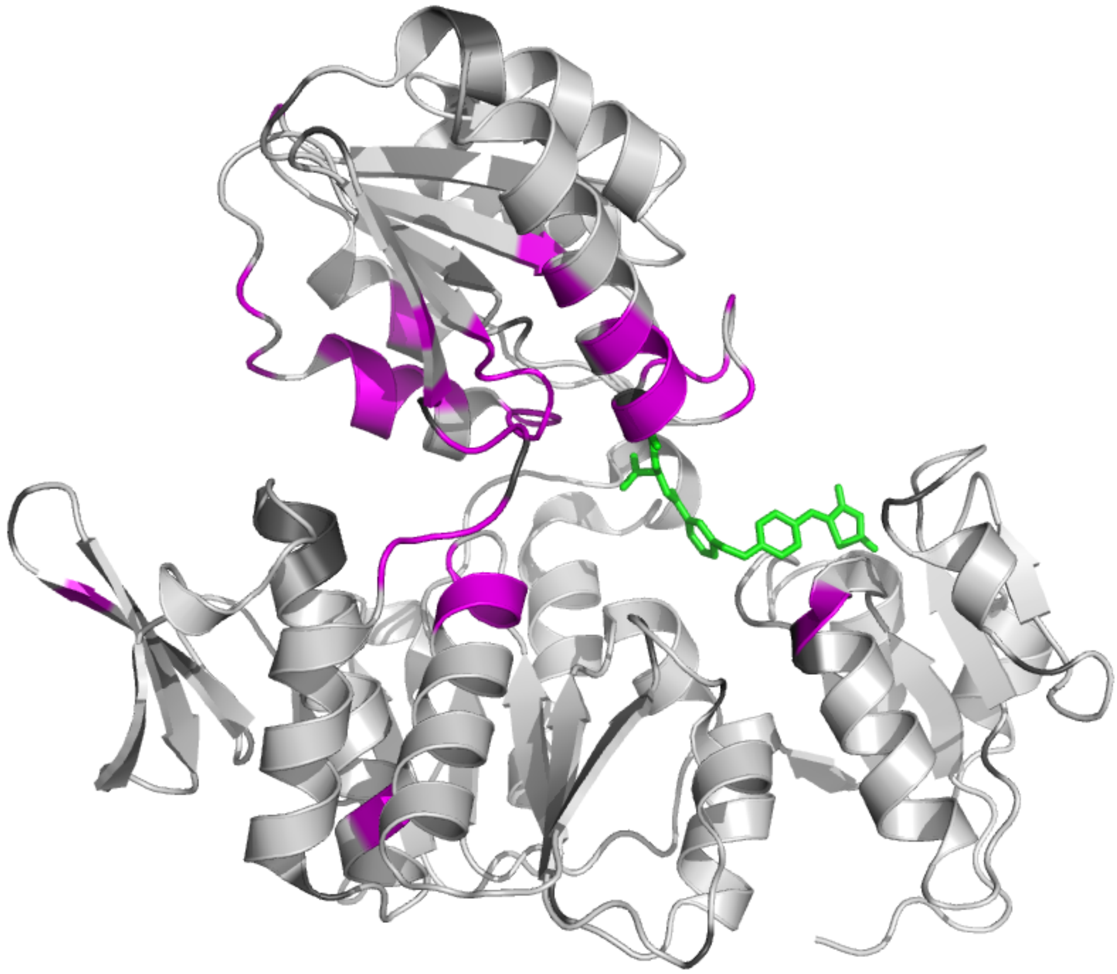

Figure S4 Mapping of the resonances that appeared by the addition of compound 1. A number of backbone amide resonances appeared by the addition of 5 equivalents of compound **1** and are colored in purple in the crystal structure of MurD in complex with compound **1** (2x5o.pdb). Unassigned residues and prolines are colored in gray. Compound 1 is represented as green sticks.

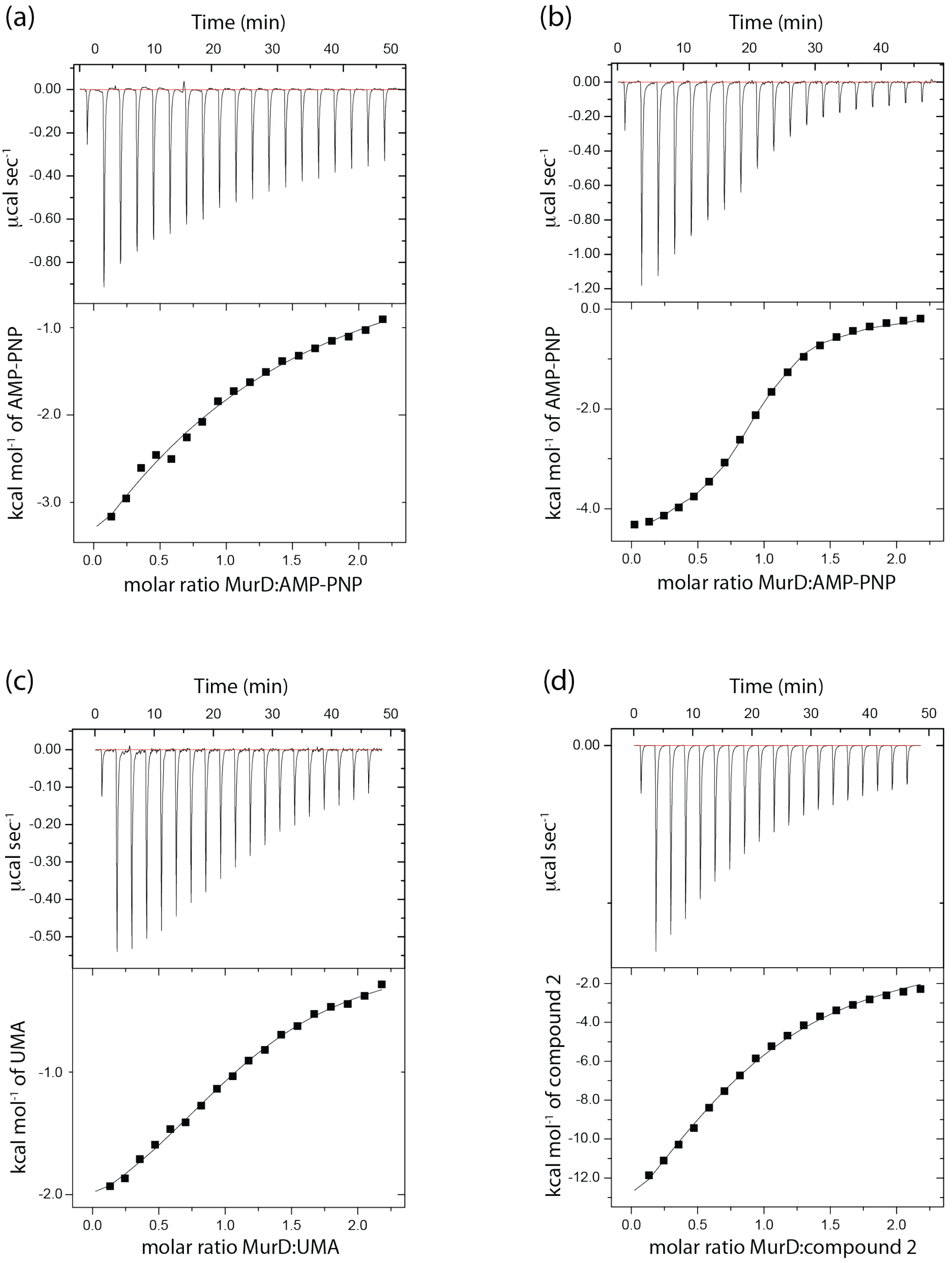

Figure S5 ITC traces of the titration of a series of the ligands to MurD. AMP-PNP (a), AMP-PNP in the presence of 5 mM Mg^{2+} (b), UMA in the presence of 5 mM Mg^{2+} and 2 mM AMP-PNP (c), and compound **2** (d) are shown.

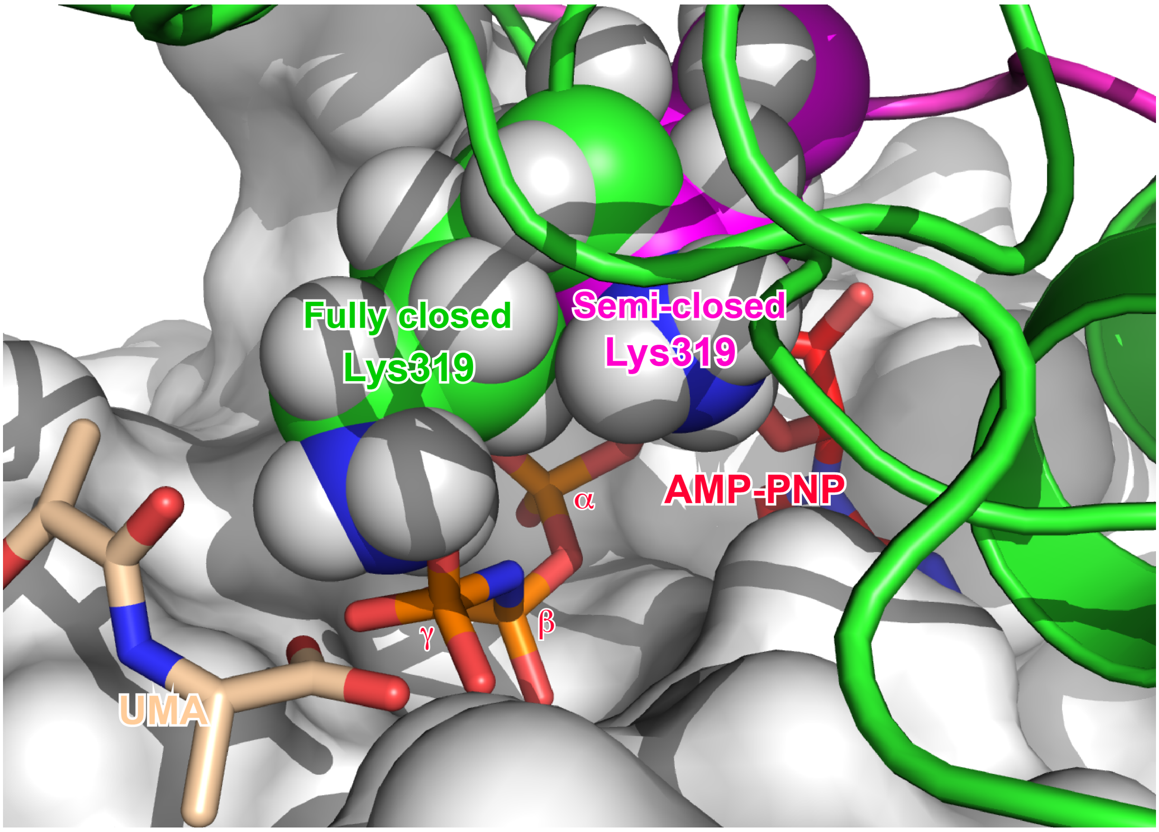

Figure S6 Close-up view of the interface between the domain 2 and 3 with semi-closed model and fully closed structure superimposed. Semi-closed model (magenta) is superimposed to the crystal structure of MurD in fully closed conformation (3uag.pdb, green). Domain 2 is represented as surface model in gray. AMP-PNP is shown as red sticks where gamma-phosphate is modeled based on the coordinates of ADP in the crystal structure (3uag.pdb) as reported by Bernt *et al*. (10). The model implies a steric clash between γ-phosphate group of AMP-PNP and ε-amino group of Lys319, while in the semi-closed conformation Lys319 moves away to make a space for the γ-phosphate. This steric clash may explain why the domain 3 remains semi-closed conformation in the presence of ATP. In the absence of γ-phosphate group (ADP-bound), ε-amino group of Lys319 locates between UMA and ADP, forming preferable hydrogen bonds with theβ-phosphate group and carboxylic tail of UMA.
